# Roles of alpha-7 nicotinic acetylcholine receptors and spleen in the lung injury induced by a repeated saline lavage in rat

**DOI:** 10.1186/s12890-022-02151-3

**Published:** 2022-09-27

**Authors:** Hossein Fatemikia, Amirreza Dehghanian, Bizhan Ziaian, Maryam Farokhipour, Farzaneh Ketabchi

**Affiliations:** 1 Department of Physiology, School of Medicine, Shiraz University of Medical Sciences, Shiraz, Iran; 2 Department of Pathology, School of Medicine, Shiraz University of Medical Sciences, Shiraz, Iran; 3 Department of Thoracic Surgery, Namazi Hospital, Shiraz University of Medical Sciences, Shiraz, Iran

**Keywords:** α7nAChR, Lung, Nicotine, MLA, Saline lavage, Spleen

## Abstract

**Background:**

The study aimed to determine whether or notα7 nicotinic acetylcholine receptors (α7nAChR) induce anti-inflammatory effects directly in the lung or through the spleen pathway in a sterile model of lung injury by saline lavage.

**Methods:**

Male Sprague Dawley rats were divided into seven groups; Sham, splenectomy (SPX), saline lavage (LAV), LAV treated with α7nAChR agonist nicotine (LAV + NIC), and LAV treated with NIC and a selective α7nAChR antagonist MLA (LAV+MLA+NIC), LAV and splenectomy (LAV+SPX), and LAV+SPX treated with nicotine (LAV+SPX+NIC). Tracheostomy and catheterization of the femoral artery were performed under deep anesthesia. Animals were subjected to volume-controlled ventilation and lung injury by 10 repeated saline lavages. Splenectomy was achieved one week before the induction of lung injury. The recovery phase lasted for 3 h, and drugs were injected 1 h after the last lavage.

**Results:**

Mean arterial blood pressure (MBP), heart rate (HR), PaO_2_, PaO_2_/FiO_2_ ratio, and pH decreased, whereas, maximal inspiratory (MIP) and expiratory (MEP) pressures, and PaCO_2_ increased 1 h after the saline lavage. Nicotine corrected entirely all the above parameters in the LAV + NIC group. MLA or SPX prevented the effects of nicotine on the above parameters, except that MLA had no extra effect on MIP or MEP. In addition, nicotine improved lung compliance in the LAV + NIC and LAV + SPX + NIC groups, though it was inhibited by MLA in the LAV + MLA + NIC group. The increases of plasma and lung tissue malondialdehyde (MDA) in the LAV group were diminished by nicotine, whereas, MLA and SPX prevented these reductions. Besides, nicotine could reduce plasma MDA in the LAV + SPX + NIC group. Total BAL cell count, protein BAL/protein plasma ratio, and lung histological scores were attenuated by nicotine in the LAV + NIC group, whereas, MLA reversed the mentioned alterations in the LAV + MLA + NIC group. However, splenectomy could not stop the decreasing effect of nicotine on the total BAL cell in the LAV + SPX + NIC group.

**Conclusions:**

In this study, we indicated that α7nAChR and spleen play roles in cholinergic anti-inflammatory pathways in saline lavage-induced lung injury. However, our results are in favor of at least some direct effects of α 7nAChR in the lung.

## Background

Acute respiratory distress syndrome (ARDS) is a serious inflammatory disorder in the lung with a high mortality rate worldwide. The immune system has been shown to play an essential role in the pathophysiology of ARDS [1, 2]. Nevertheless, no effective therapeutic approach to the disease still exists [3, 4]. Over the past two decades, the cholinergic therapeutics are reported to alleviate inflammation in the body’s organs [5–7]. Carbachol and nicotine have been indicated to induce anti-inflammatory effects through α7 nicotinic acetylcholine receptors (α7nAChR) in lipopolysaccharide (LPS)-induced endotoxemia [8]. α7nAChR is expressed in alveolar macrophages, lung epithelial cells, and human venous endothelial cells [9, 10]. Activation of α7nAChR by nicotine alleviates lung injury induced by LPS or Escherichia coli, while α7nAChR deficiency develops severe lung injury [11, 12]. Specific and non-specific α7nAChR agonists attenuate lung injury induced by mechanical ventilation or intra-tracheal instillation of hydrochloride acid, which is prevented by administration of α7nAChR antagonist [9, 13, 14]. In addition, vagal stimulation has been demonstrated to be effective in alleviating high-tidal volume and ischemia/reperfusion lung injuries, whereas, bilateral vagotomy has the opposite effect [13].

Some studies have reported controversial findings regarding the role of the vagus nerve in lung inflammation. This may be related to the differences between experimental models used for the induction of acute lung injury [15]. For instance, stimulation of the vague nerve is not beneficial in a double hit model of lung injury by LPS with mechanical ventilation, whereas α7nAChR agonists diminish lung injury induced by LPS or mechanical ventilation [12, 14, 16]. Furthermore, nicotine weakens the host defense in pneumonia, while it is unable to inhibit the inflammatory response to gram-positive streptococcus in the lung or circulation [17]. Activation of α7nAChR may also differently affect the injured lung depending on the pattern recognition receptors [15]. Toll-like receptor 4 (TLR_4_) recognizes gram-negative LPS, whereas, TLR_2_ identifies gram-positive components [18]. The protective effect of α7nAChR occurs downstream of TLR4, with no effect through TLR2 [11].

Spleen has a significant role in the cholinergic anti-inflammatory pathways (CAP) [19]. The preganglionic parasympathetic neurons stimulate the postganglionic sympathetic neurons in the celiac plexus. The latter activates β-adrenergic receptors in the spleen-resident T-cells and leads to acetylcholine (ACh) release. ACh stimulates α7nAChR on macrophages, where it decreases cytokines release and inflammatory reactions [19]. Some reports indicated that spleen diminishes lung injury induced by renal ischemia/reperfusion injury by elevating anti-inflammatory cytokines [20]. In addition, spleen prevents the activation of inflammatory macrophages in the lung injury induced by nitrogen mustard [21]. In contrast, splenectomy inhibits cytokine production and oxidative stress in lung injury following intestinal ischemia/reperfusion [22]. Together, a question remains whether or not the activation of α7nAChR induces anti-inflammatory effects directly in the lung or through the spleen pathway.

ARDS in infants and adults may be associated with surfactant abnormalities such as reduced surfactant production, surfactant dysfunction and degradation, and its incorporation to hyaline membrane [23, 24]. Surfactant depletion by saline lavage is a reproducible and sterile model of lung injury in animals [25, 26]. Some of the fundamental features of acute lung injury in human including histopathological alterations in the lung, disruption of the alveolar capillary barrier, inflammatory criteria, and impairment of gas exchange have been reported in this model of lung injury [24, 25, 27–31]. As a result, lung injury induced by saline lavage can be used as a valuable way to investigate the pathophysiology and therapeutic approaches of lung injury in humans.

With the above background, in this study, we investigated the effects of α7nAChR agonist and antagonist in the presence or absence of spleen in the lung injury induced by repeated saline lavages in rat. Since the cholinergic pathways could influence the function of the cardiovascular system, we assessed systemic blood pressure and heart rate alongside with other parameters of lung injury and lung compliance at the above conditions.

## Materials and methods

Male Sprague–Dawley rats (n = 77, body weight:2.95 ± 2.62 g) were housed in the animal house of the department of physiology kept in standard cages, controlled temperature (24–26ºC), humidity (60–65%), and 12:12 h of light/dark cycles, and had free access to water and food days before the study.

### Study protocol and design

**Fig. 1:**
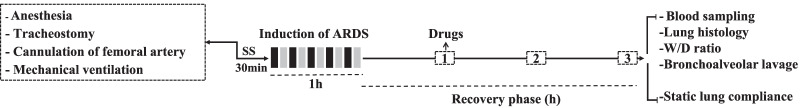
Representative timeline of the study design during 1 h (h) of broncoalveolar lavage and 3 h of recovery phase. *SS* Steady-state period

Animals underwent anesthesia by 60 mg/kg of sodium thiopental (VUAB Pharma Inc. Czech Republic). Additional doses were used when appropriate. The body temperature of animals was maintained in the range of 37 ± 0.5 °C. After tracheostomy, a heparinized tubing cannula (PE-50) was inserted into the femoral artery and connected to a data acquisition system (Power Lab, ML 786, AD instruments, Australia) through a pressure transducer. A blood sample (0.3 ml) was taken for blood gas analysis. The tracheal tube was connected to a rodent ventilator (Palmer, England). Volume-controlled ventilation was started with a fraction of inspired oxygen (FiO_2_) of 0.5, tidal volume of 12 mg/kg, and respiratory rate of 50 breaths/min. The inspiratory and expiratory tubing was connected to the Power Lab system (MLT0699, AD instruments, Australia) through pressure transducers. The mean arterial blood pressure (MBP), heart rate (HR), maximum inspiratory airway pressure minus PEEP (MIP), and maximum expiratory airway pressure minus PEEP (MEP) were continuously recorded throughout the experiments. Animals were divided into seven groups: Sham, splenectomy (SPX), saline lavage-induced lung injury (LAV), LAV treated with α7nAChR agonist nicotine (LAV + NIC), LAV treated with NIC and a selective α7nAChR antagonist methyllycaconitine (LAV+MLA+NIC), LAV and splenectomy (LAV+SPX), and LAV+SPX treated with nicotine (LAV+SPX+NIC). N = 10–12 in each group. One experiment per each group was performed randomly in a single week. After 30 min of the steady-state period, lung injury was induced by 10 repeated saline lavages with 30 ml saline at 37 °C (3 ml in each lavage) and time intervals of 5 min. To lessen barotrauma, positive end-expiratory pressure (PEEP) was raised serially 2 min before the first four lavages. PEEP was then fixed at 4 Cm H_2_O (⁓3 mm Hg), being constant until the end of the experiments. The arterial blood samples were taken and analyzed by an Easy Blood Gas analyzer (Medica, USA) during a steady-state period, and 1, 2, and 3 h after the last lavages. One hour after the last lavage, animals with an arterial oxygen pressure (PaO_2_) of less than 100 mm Hg were included in the study. Also, data from a few animals which were died during the experiments were excluded from the study (Sham = 0, LAV = 3, LAV + NIC = 1, LAV + MLA + NIC = 3, SPX = 0, LAV + SPX = 5, LAV + SPX + NIC = 2). One hour after the last lavage, 0.4 mg/kg of nicotine (Sigma, Germany) was injected i.p [12, 17]. Also, 5 min before the injection of nicotine, 1 mg/kg of MLA (Sigma, Germany) was injected i.p in the LAV + MLA + NIC group [14]. At the end of the experiments, a blood sample was taken for blood cell count and blood gas analyses. Animals were sacrificed under deep anesthesia and divided into two subgroups (n = 5–6 in each subgroup) to minimize the effects of procedures on the measurements. In the first subgroup, the chest was opened and the hilum of the right lung was closed with a suture to separate it from the left lung. The upper lobe of the right lung was removed, washed with PBS, weighted, and kept at room temperature. After 5 days, the wet/dry weight ratio (W/D ratio) was calculated. The middle lobe of the right lung was stored at − 70 °C to measure malondialdehyde (MDA), as an indicator of lipid peroxidation and oxidative stress. The lower lobe of the right lung was placed in 4% formalin for H&E staining and histological assessments. Bronchoalveolar lavage (BAL) was then achieved in the left lung. In the second subgroup, the static lung compliance was evaluated. Figure 1 shows a representative timeline of the study design.

### Splenectomy

Splenectomy was performed one week before the induction of lung injury to minimize the stress induced by surgery in the second phase of the experiments. Animals were anesthetized with an intraperitoneal injection of ketamine (80 mg/kg) and xylazine (10 mg/kg). Body temperature was monitored via a rectal probe and maintained at 37 ± 0.5 °C during the surgery. After a small incision in the skin and abdominal muscles at the left side of the abdomen, the splenic arteries were ligated, the spleen removed with caution, and the skin and abdominal muscles closed with 4–0 silk suture [32]. In the end, the conscious animals were returned to the separated clean cages in the animal house and remained in the vivarium until the day of the experiments. All surgical procedures were performed under aseptic conditions.

### Static lung compliance

After sacrificing the animals, the chest was opened at midline. The rib cages were pushed aside to prevent any lung damage. The ventilator was separated from the animals. A stopcock was connected to the trachea. A pressure transducer and a 50 ml air-filled syringe were connected to the other sides of the stopcock. Air was gently (5 s) injected (2 ml each time) into the animal's lungs. The airway pressure was allowed to be stabilized for another 5 s. The equilibrium airway pressure was recorded at each step. The injection of air continues until the airway pressure reached about + 30 mm Hg. The same procedure was performed for air removal [5-s deflation (2 ml air), 5-s equilibration]. The pressure–volume curve was then depicted during inflation and deflation maneuvers [33].

### Cell counts and protein concentration in the BAL fluid and blood

Two ml of warm saline (37 °C) was injected through the trachea into the left lung. After 30 s, it was gently aspirated. This procedure was repeated three times. The aspirated volume of more than 80% of BAL fluid was considered for the study. BAL fluid was then centrifuged at 1500 rpm for 10 min at 4 °C and the supernatant was kept at – 20 °C to measure protein concentration. 0.5 ml of PBS was added to the precipitated cells and shaken slowly to disperse the cells in a homogeneous liquid. Then, 50 μl of the liquid was diluted with 50 μl of Marcano solution. Also, 10 μl of blood was diluted with 190 μl of Marcano solution. The total number of leukocytes in the BAL and blood were then counted using a hemocytometer and a microscope (Olympus, Japan) with 10× magnification. Differential types of white blood cells were also determined by staining the smear of blood and BAL with wright’s solution. 100 cells were counted using the microscope with 100× magnification and the percentage of leukocyte types was calculated. Protein concentration in the blood and the BAL were measured using the digital photometric method. The ratio of protein concentration in BAL/protein concentration in plasma ([Pr] BAL/[Pr] plasma ratio) was then calculated.

### Lung histology

The slide preparations and staining of lungs with hematoxylin and eosin were performed in the department of pathology. All slides of histology were evaluated in a blinded manner by a pathologist using six pathological indexes of perivascular hemorrhage, perivascular edema, alveolar hemorrhage, infiltration of neutrophils, alveolar membrane, and alveolar edema [34].

### Plasma and lung tissue MDA

Tissue samples were stored at −70 °C until the day of the measurements. 300 μl of KCl (1.15%) was added to 50 mg of the lung tissue sample in 2 ml aliquot homogenized for 4 min. 100 μl of SDS (8%) was added to the suspended solution and kept for 10 min  in ice. Then, it was centrifuged at 12,000 rpm for 20 min at 4 °C and the supernatant was collected. Plasma and lung MDA was determined using a TBARS assay. Briefly, 100 μl of plasma, supernatant samples, or standard solutions (1, 1,3,3-tetraethoxy propane, Merck, Germany) was mixed with 400 μl of combinations of 0.25 N, HCl (Sigma, Germany), 20% trichloroacetic acid (Sigma, Germany) and 0.8% tribarbituric acid (Sigma, Germany), incubated at 90 °C for 60 min, cold in ice, and centrifuged at 4000 rpm for 10 min. Then, 200 μl of prepared solutions was added to each well, and the absorbance of the well-plate was read at 532 nm using a microplate reader (Biotek, USA) [35].

### Statistical analysis

All values and parameters were measured or calculated based on mean ± SD using SPSS 23. Shapiro–Wilk test was used to determine the normal distribution of data. The values of MBP, HR, MIP, and MEP at baseline, and lung compliance, lung and plasma MDA, [Pr] BAL/[Pr] plasma ratio, lung W/D ratio, and cell counts in BAL and blood had normal distribution. These parameters were analyzed by a one-way analysis of variance (ANOVA) with Tukey post-test. Post lavage parameters of MBP, HR, MIP, MEP had normal distribution and were analyzed by repeated measured ANOVA. Intergroup analysis was performed using a paired-t-test. Data of blood gas parameters and lung histology were assessed using nonparametric Kruskal–Wallis tests and Bonferroni-corrected Mann–Whitney U test. A significant difference was assumed when *P* < 0.05 and the confidence limits used were the 95% intervals.

## Results

### MBP and HR decreased in all lavage groups

**Fig. 2:**
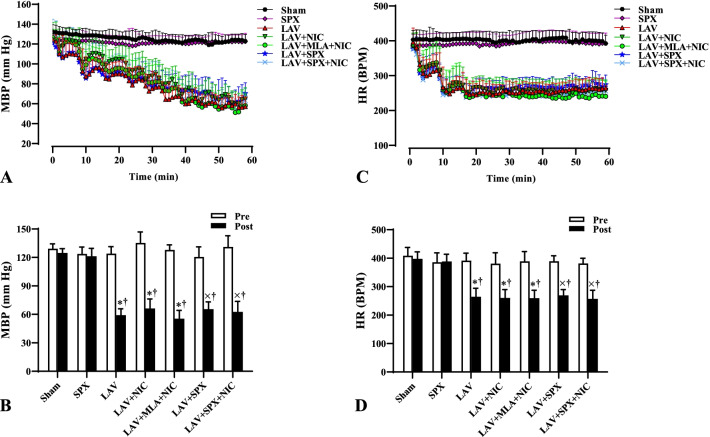
Alterations of mean arterial blood pressure (MBP, **A**) and heart rate (HR, **C**) during 60 min’ induction of lung injury in all lavage groups, and with equal duration in the Sham and SPX groups. The mean values of MBP (**B**) and HR (**D**) were compared 5 min before the starting point (pre) and 5 min after the 10th lavage (post), and with the same duration in the Sham and SPX groups without lavage. n = 10 in each group. Data are mean ± SD. *: *P* < 0.05, each group versus the Sham group; ×: *P* < 0.05, each group versus the SPX group; †: *P* < 0.05, the post values versus the pre values. *LAV* Lavage; *NIC* Nicotine; *SPX* Splenectomy; *MLA* Methyllycaconitine

Figure 2A and C reveal alterations in mean arterial blood pressure (MBP) and heart rate (HR) during ten lavages (60 min), and with the same duration in the Sham and SPX groups. In all lavage groups, MBP started to decrease slowly, reaching minimum values after the 10th lavage. Also, HR was sharply diminished during the 1st-3th lavages and then remained stable at minimum values until the last lavages. We compared MBP and HR at 5 min before the first lavage (pre) and 5 min after the 10th lavage (post), and with equal duration in the Sham and SPX groups in Fig. 2B and D. There was no a difference in basal (pre) values of MBP and HR between all groups. After the last lavage, MBP and HR were lower than their baseline values, in the LAV, LAV + NIC, and LAV + MLA + NIC groups were less than in the Sham group, and in the LAV + SPX and LAV + SPX + NIC groups were less than in the SPX group.

### MIP and MEP increased in all lavage groups

**Fig. 3:**
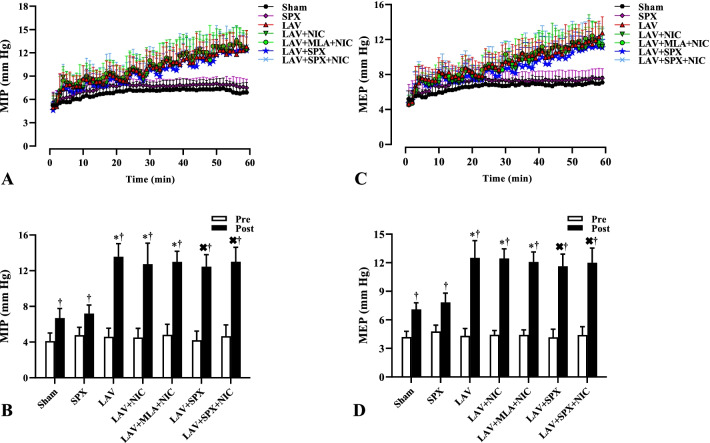
Alterations of maximum inspiratory pressure (MIP, **A**) and maximum expiratory pressure (MEP, **C**) during 60 min’ induction of lung injury, and with equal duration in the Sham and SPX groups. The mean values of MIP (**B**) and MEP (**D**) were compared 5 min before the starting point (pre) and 5 min after the 10th lavage (post), and with the same duration in the Sham and SPX groups without lavage. n = 10 in each group. Data are mean ± SD. *: *P* < 0.05, each group versus the Sham group; ×: *P* < 0.05, each group versus the SPX group; †: *P* < 0.05, the post values versus the pre values. *LAV* Lavage; *NIC* Nicotine; *SPX* Splenectomy; *MLA* Methyllycaconitine

Figure 3A and C indicate alterations in maximum inspiratory pressure (MIP) and maximum expiratory pressure (MEP), and with the same duration as alterations in MBP and HR. MIP and MEP increased progressively from the 1st lavage to the 10th lavage. Furthermore, MIP and MEP little increased during the first 20 min in the Sham and SPX groups and then remained stable until the end of the experiments. We compared the mean values of MIP and MEP at 5 min before the first lavage and 5 min after the 10th lavage, and with equal duration in the Sham and SPX groups in Fig. 3B and D. There was no a difference in basal (pre) values of MIP and MEP between all groups. After the 10th lavage, MIP and MEP in all groups were more than their baselines. Furthermore, MIP and MEP in the LAV, LAV + NIC and LAV + MLA + NIC groups were more than in the Sham group, and in the LAV + SPX and LAV + SPX + NIC groups were more than in the SPX group.

### Administration of nicotine returned partly blood gas parameters to normal values during the recovery phase

**Fig. 4:**
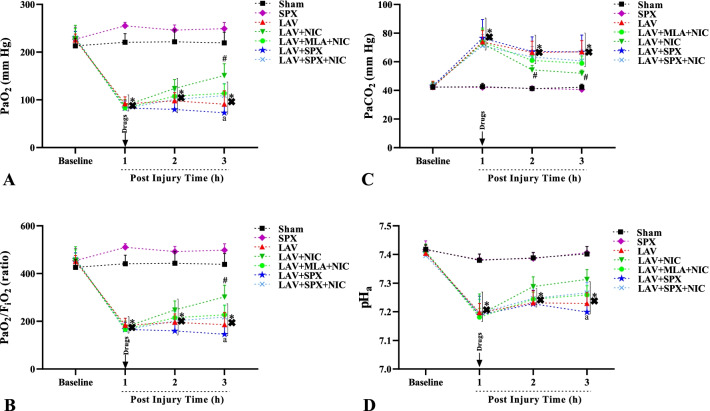
PaO_2_ (**A**), PaO_2_/FiO_2_ (**B**), PaCO_2_ (**C**), and pHa (**D**) in the arterial blood at baseline, and 1, 2, and 3 h of the recovery phases in lavage groups, and with the same duration in the Sham and SPX groups. n = 10 in each group. Data are mean ± SD. *: *P* < 0.05, each group versus the Sham group; ×: *P* < 0.05, each group versus the SPX group; #: *P* < 0.05, each group versus the LAV group; a: *P* < 0.05, the LAV + SPX group versus the LAV + NIC group. *LAV* Lavage; *NIC* Nicotine; *SPX* Splenectomy; *MLA* Methyllycaconitine

Figure 4A–D indicate PaO_2_, PaO_2_/FiO_2_ ratio, PaCO_2_, and pH at baselines and 1, 2, and 3 h after the last lavages (recovery phases), and with the same duration in the Sham and SPX group. There was no a difference in basal (pre) values of the above parameters between all groups. After 1 h of the recovery phase, PaO_2_, PaO_2_/FiO_2_ ratio, and pH decreased, and PaCO_2_ increased in all lavage groups. Even after 2 h, PaO_2_, PaO_2_/FiO_2_ ratios, and pH in all lavage groups were similarly lower than in the Sham and SPX groups. However, nicotine corrected PaO_2_ and PaO_2_/FiO_2_ ratio after 3 h and improved PaCO_2_ after 2 and 3 h of the recovery phase in the LAV + NIC group compared with the LAV group. MLA prevented the corrections in PaO_2_, PaO_2_/FiO_2_ ratio and PaCO_2_ in the LAV + MLA + NIC group. All mentioned data in the LAV + MLA + NIC group were not different from the LAV group. After 1, 2, and 3 h of the recovery phase, there was no a significant difference in PaO_2_, PaO_2_/FiO_2_ ratio, PaCO_2_, and pH between the LAV + NIC and LAV + MLA + NIC groups. Although pH tended to increase during the recovery phase in the LAV + NIC group, data was not statistically different compared to the LAV group. Splenectomy did not change all mentioned parameters in the LAV + SPX group compared with the LAV group. After 3 h of the recovery phase, PaO_2_, PaO_2_/FiO_2_ ratio, and pH in the LAV + SPX group were lower than in the LAV + NIC group. In addition, nicotine could not correct these parameters in the LAV + SPX + NIC group significantly.

### Alterations of MBP, HR, MIP, and MEP during the recovery phase

**Fig. 5:**
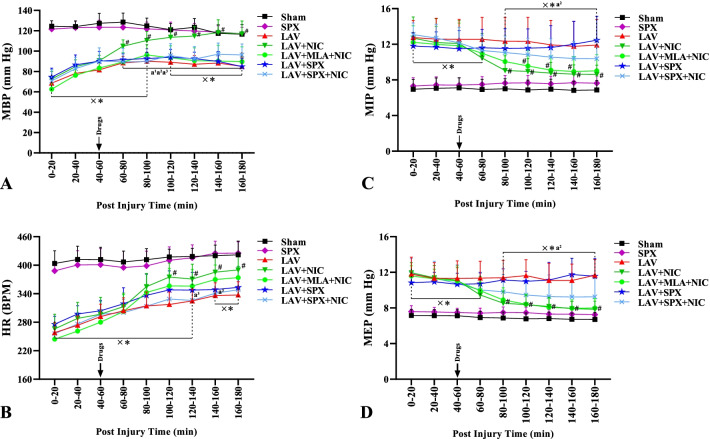
Alterations of arterial pressure (MBP, **A**), heart rate (HR, **B**), maximum inspiratory pressure (MIP, **C**), and maximum expiratory pressure (MEP, **D**) during 3 h of the recovery phase, and with the same duration in the Sham and SPX groups. n = 10 in each group. Data are mean ± SD. *: *P* < 0.05, each group versus the Sham group; ×: *P* < 0.05, each group versus the SPX group; #: *P* < 0.05, each group versus the LAV group; a^1^: *P* < 0.05, the LAV + MLA + NIC group versus the LAV + NIC group; a^2^: *P* < 0.05, the LAV + SPX group versus the LAV + NIC group; a^3^: *P* < 0.05, the LAV + SPX + NIC group versus the LAV + NIC group. *LAV* Lavage; *NIC* Nicotine; *SPX* Splenectomy; *MLA* Methyllycaconitine

Figure 5A–D reveal the mean values of MBP, HR, MIP, and MEP during 3 h of the recovery phase in all lavage groups, and with the same duration in the Sham and SPX groups. MBP increased slowly in all lavage groups, though they were lower than the Sham and SPX groups during 0–100 min of the recovery phase. Only in the LAV + NIC group, it reached the values in the Sham group during 100–180 min. In addition, MBP in the LAV + NIC group was higher than the LAV group during 60–180 min. MLA prevented MBP to increase in the LAV + MLA + NIC group. As a result, MBP in the LAV + MLA + NIC group was lower than in the LAV + NIC group during 60–180 min. Splenectomy in the LAV+SPX group and nicotine in the LAV+SPX+NIC group did not affect MBP compared with the LAV group. In addition, MBP in the LAV + SPX and LAV + SPX + NIC groups were lower than in the LAV + NIC group during 60–180 min.

HR increased progressively in the recovery phase of all lavage groups. HR in the LAV + NIC group was lower than the Sham group during 0–140 min reached the values in the Sham group during 140–180 min of the recovery phase. Furthermore, HR in the LAV + NIC group were more than in the LAV group during 100–180 min. HR in the LAV + MLA + NIC group was lower than the Sham group during 0–140 min significantly and during 140–180 min insignificantly. No significant alteration was detected in HR between the LAV + MLA + NIC and LAV + NIC groups. HR in the LAV+SPX and LAV+SPX+NIC groups were similarly lower than the SPX group during 0–180 min, and did not differ compared with the LAV group. In addition, HR in the LAV + SPX + NIC group were less than the LAV + NIC group during 120–160 min. Also, the difference in HR between the LAV + SPX + NIC and the LAV + MLA + NIC groups was not remarkable.

MIP and MEP in all lavage groups were more than the Sham and SPX groups during 0–80 min of the recovery phase. Nicotine decreased remarkably MIP and MEP in the LAV + NIC group reached the values lower than the LAV group during 80–180 min. MIP in the LAV + MLA + NIC group were less than the LAV group during 100–160 min. Also, MEP in the LAV + MLA + NIC group were less than the LAV group during 80–180 min. There was no alteration in MEP and MIP between the LAV + NIC and LAV + MLA + NIC groups during 0–180 min. MIP and MEP did not decrease in the LAV + SPX group compared with the LAV group. Also, nicotine in the LAV + SPX + NIC group could not change these values significantly. MIP and MEP in the LAV + SPX group   were more than in the SPX group during 0–180 min. Furthermore, MIP and MEP in the LAV + SPX group were more than the LAV + NIC group during 80–180 min. There was no a difference in MIP and MEP between the LAV + SPX + NIC and LAV + NIC groups.

### Nicotine increased lung compliance in the lavage groups

**Fig. 6:**
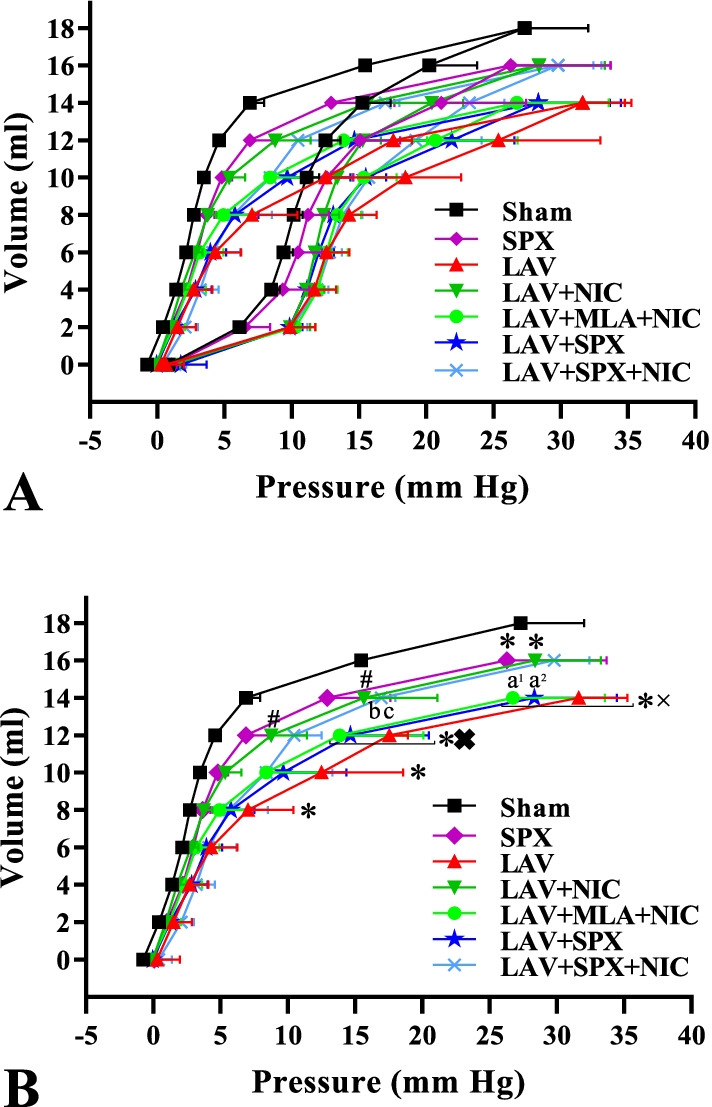
Pressure–volume curves in the inflation and deflation maneuvers (**A**) and only in the deflation maneuver (**B**) in the Sham (n = 5), SPX (n = 5), LAV (n = 6), LAV + NIC (n = 5), LAV + MLA + NIC (n = 6), LAV + SPX (n = 5), and LAV + SPX + NIC (n = 5) groups. Data are mean ± SD. *: *P* < 0.05, each group versus the Sham group; ×: *P* < 0.05, each group versus the SPX group; #: *P* < 0.05, each group versus the LAV group; a^1^: *P* < 0.05, the LAV + MLA + NIC group versus the LAV + NIC group; a^2^: *P* < 0.05, the LAV + SPX group versus the LAV + NIC group; b: *P* < 0.05, the LAV + SPX + NIC group versus the LAV + MLA + NIC group; c: *P* < 0.05, LAV + SPX + NIC group versus the LAV + SPX group. *LAV* Lavage; *NIC* Nicotine; *SPX* Splenectomy; *MLA* Methyllycaconitine

Figure 6A indicates the pressure–volume curves during inflation and deflation maneuvers. Only deflation maneuver is depicted and analyzed in Fig. 6B. The decrease in lung compliance in the LAV group was detected compared with the Sham group in deflation volume of 8 ml to the higher deflation volumes. Nicotine could partially improve lung compliance: in deflation volumes of 12 and 14 ml in the LAV + NIC group, it was more than in the LAV group. The deflation volume of 16 ml was only detected in the LAV+NIC group but not in the LAV group. Furthermore, in deflation volume of 16 ml, lung compliance in the LAV + NIC group was lower than in the Sham group. MLA prevented the increase in lung compliance: in deflation volumes of 12 and 14 ml, in the LAV + MLA + NIC group it was lower than the Sham group. Furthermore, in deflation volume of 14 ml, lung compliance in the LAV + MLA + NIC group was lower than in the LAV + NIC group. There was no a significant difference in lung compliance between the LAV + MLA + NIC and LAV groups at any deflation volume.

In deflation volume of 16 ml, the lung compliance in the SPX group was lower than the Sham group. In deflation volumes of 12 and 14 ml, the lung compliance in the LAV + SPX group was lower than the SPX group. There was no a significant difference in lung compliance between the LAV + SPX and LAV groups at any deflation volume. However, the lung compliance in the LAV + SPX + NIC group was more than the LAV + SPX and LAV + MLA + NIC groups in deflation volume of 14 ml.

### Lipid peroxidation and vascular permeability in the experimental groups

**Fig. 7:**
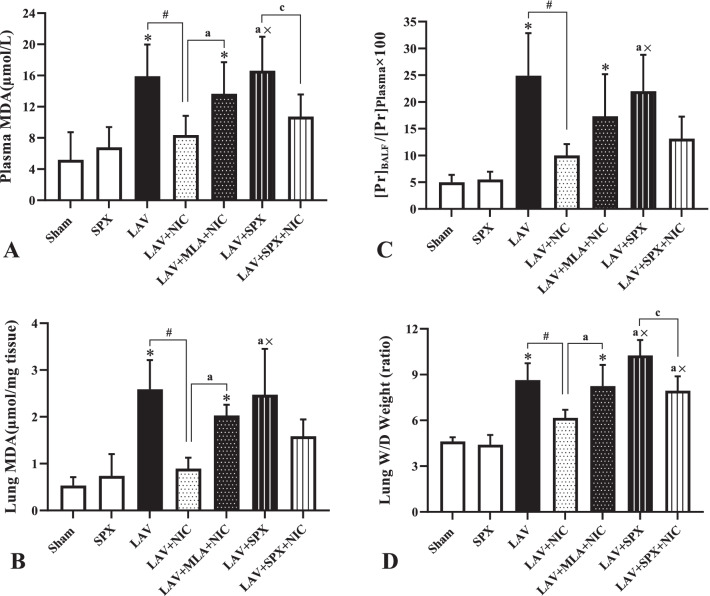
Plasma MDA (n = 10 in each group, **A**), and lung tissue MDA (**B**), [pr] BAL/ [pr] plasma ratio (**C**), and lung wet/dry (W/D) weight ratio  (**D**) in the Sham (n = 5), SPX (n = 5), LAV (n = 6), LAV + NIC (n = 6), LAV + MLA + NIC (n = 6), LAV + SPX (n = 6), and LAV + SPX + NIC (n = 6) groups. Data are mean ± SD. *: *P* < 0.05, each group versus the Sham group; ×: *P* < 0.05, each group versus the SPX group; #: *P* < 0.05, each group versus the LAV group; a: *P* < 0.05, each group versus the LAV + NIC group; c: *P* < 0.05, each group versus the LAV + SPX group. *LAV* Lavage; *NIC* Nicotine; *SPX* Splenectomy; *MLA* Methyllycaconitine

Data of plasma and lung MDA are indicated in Fig. 7A and B. The values of MDA in plasma and lung tissue in the LAV group were higher than those in the Sham group. Both parameters decreased significantly in the LAV + NIC group compared with the LAV group. MLA increased MDA in plasma and lung: in the LAV + MLA + NIC group, they were significantly more than those in the LAV + NIC group. The level of plasma and lung MDA in the LAV + SPX group was more than the SPX and LAV + NIC groups. Plasma MDA in the LAV + SPX + NIC group decreased compared with the LAV + SPX group. However, the reduction of MDA in the lung of the LAV + SPX + NIC group was not significant.

Protein concentration in BAL/protein concentration in plasma ratio ([Pr] BAL/[Pr] plasma ratio) and lung wet/dry (W/D) weight ratio measured to evaluate pulmonary vascular permeability. [Pr] BAL/[Pr] plasma ratio in Fig. 7C increased in the LAV group, attenuated by nicotine in the LAV + NIC group, and increased by MLA in the LAV + MLA + NIC group. However, there was no a significant difference in the ratio between the LAV + NIC and LAV + MLA + NIC groups. [Pr] BAL/[Pr] plasma ratio increased in the LAV + SPX group: in this group it was more than in the SPX and LAV + NIC groups. Although, this ratio tended to decrease in the LAV + SPX + NIC group, it was not significant.

The W/D ratio in Fig. 7D in the LAV group was higher than the Sham group. It significantly decreased in the LAV + NIC group. In the LAV + MLA + NIC group, it was significantly more than the LAV + NIC and Sham groups. The ratio in the LAV + SPX group was more than the SPX and LAV + NIC groups. Also, it decreased in the LAV + SPX + NIC group compared with the LAV + SPX group. However, the ratio in the LAV + SPX + NIC group was still more than the SPX and LAV + NIC groups.

### Lung histological score in the experimental groups

**Fig. 8:**
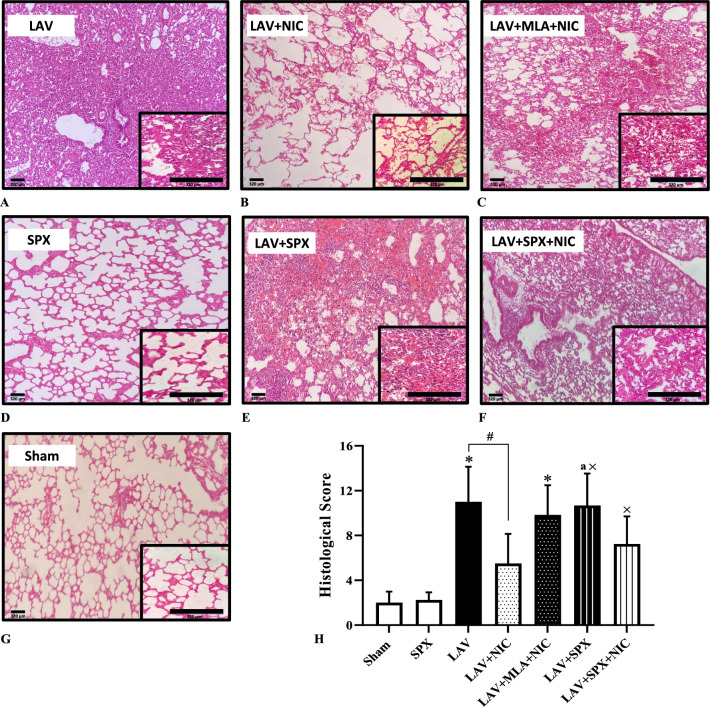
Representative lung hematoxylin eosin staining at 100 and 400 magnifications (**A**-**G**) and the lung histopathological score in the Sham (n = 5), SPX (n = 5), LAV (n = 6), LAV + NIC (n = 6), LAV + MLA + NIC (n = 6), LAV + SPX (n = 6), and LAV + SPX + NIC (n = 6) groups (**H**). Scale bars are indicative of 320 μm. Data are mean ± SD. *: *P* < 0.05, each group versus the Sham group; ×: *P* < 0.05, each group versus the SPX group; #: *P* < 0.05, each group versus the LAV group; a: *P* < 0.05, each group versus the LAV + NIC group. *LAV* Lavage; *NIC* Nicotine; *SPX* Splenectomy; *MLA* Methyllycaconitine

The representative lung hematoxylin eosin staining in all experimental groups indicated in the Fig. 8A–G. The lung histological score in Fig. 8H increased in the LAV group, attenuated by nicotine in the LAV + NIC group, and increased by MLA in the LAV + MLA + NIC group (insignificant compared with the LAV + NIC group). The histological score in the LAV + SPX group was more than the SPX and LAV + NIC groups, and decreased in the LAV + SPX + NIC group, though it was still more than the score in the SPX group.

### Alterations of immune cells in blood and BAL

Table 1 indicates the total and differential WBC obtained from the blood at the end of the experiments. Total WBC in the LAV group was remarkably lower than in the Sham group. Nicotine increased WBC in the LAV + NIC group compared with the LAV group, which was almost equal to the values in the Sham group. In the LAV group, neutrophils % was lower and lymphocytes % was more than those in the Sham group, whereas, nicotine in the LAV + NIC group brought data close to data in the Sham group. Furthermore, MLA prevented these alterations in the LAV + MLA + NIC group. There was no a significant alteration in total and differential WBC between the LAV and LAV + MLA + NIC groups. Total WBC in the LAV + SPX group was remarkably lower than in the SPX and LAV + NIC groups. Nicotine increased WBC in the LAV + SPX + NIC group compared to the LAV + SPX group. In the LAV + SPX group, neutrophils % was lower, and lymphocytes % was more than SPX and LAV + NIC groups. In the LAV+SPX+NIC group, the neutrophils % was lower than the SPX group and more than the LAV + SPX group. Also, in the  LAV+SPX+NIC group, lymphocytes % was more than the SPX and less than the LAV + SPX group.

**Table 1:** Total and differential WBC taken from the blood at the end of experiments

	Total WBC / (mm^3^)	Neutrophil (%)	Lymphocyte (%)	Basophils (%)	Eosinophil (%)	Monocyte (%)
Sham	8340 ± 2552	64.50 ± 6.45	28.70 ± 7.11	1.80 ± 0.78	1.90 ± 0.87	3.00 ± 1.24
SPX	9443 ± 1926	69.00 ± 8.24	25.00 ± 7.61	0.82 ± 0.51	3.00 ± 2.13	2.37 ± 1.06
LAV	5825 ± 2585*	49.40 ± 13.17*	45.30 ± 13.69*	1.40 ± 0.51	1.80 ± 0.91	2.20 ± 1.03
LAV + NIC	8290 ± 1884#	60.00 ± 7.70	35.50 ± 7.86	1.10 ± 0.87	1.30 ± 0.48	2.20 ± 1.03
LAV + MLA + NIC	7445 ± 1038	50.00 ± 7.74*	45.30 ± 8.32*	1.10 ± 0.56	1.50 ± 0.52	2.10 ± 1.10
LAV + SPX	4310 ± 1157×a	42.80 ± 4.46×a	53.01 ± 5.54×a	1.20 ± 0.63	2.90 ± 1.52	2.20 ± 0.78
LAV + SPX + NIC	8995 ± 809c	56.50 ± 7.94×c	38.80 ± 7.92×c	0.80 ± 0.42	1.80 ± 0.78	2.10 ± 0.56

Data are mean ± SD. n = 10 in each group

*LAV* Lavage, *NIC* Nicotine, *SPX* Splenectomy, *MLA* Methyllycaconitine

*: *P* < 0.05, versus the Sham group; **#**: *P* < 0.05, versus the LAV group; a: *P* < 0.05, versus the LAV + NIC group; c: *P* < 0.05, versus the LAV + SPX group; ×: *P* < 0.05, versus the SPX group

In Table 2, total BAL cell count increased in the LAV group, partly attenuated by nicotine in the LAV + NIC group, and increased in the LAV + MLA + NIC group. In the LAV group, neutrophils % increased and macrophages decreased compared to the Sham group. Nicotine decreased neutrophils % and increased macrophages % in the LAV + NIC group, whereas MLA in the LAV + MLA + NIC had the opposite effects. Total WBC and neutrophils % in the LAV + MLA + NIC group were more, and macrophages % was lower than those in the Sham and LAV + NIC groups. Total WBC and neutrophils % in the LAV + SPX group were more, and macrophages % was lower than those in the SPX and LAV + NIC groups. Nicotine in the LAV + SPX + NIC group partially decreased total WBC and neutrophils % and increased macrophages %.

**Table 2:** Total WBC, neutrophils %, and macrophages % in bronchoalveolar lavage (BAL) taken at the end of experiments

	Total cells (Cells.mm^−3^)	Neutrophils (%)	Macrophages (%)
Sham	384 ± 98	27.00 ± 2.73	72.60 ± 3.20
SPX	379 ± 116	22.80 ± 7.91	77.20 ± 7.91
LAV	1086 ± 147*	77.50 ± 3.61*	22.83 ± 4.99*
LAV + NIC	735 ± 127*#	44.33 ± 6.08*#	55.66 ± 6.08*#
LAV + MLA + NIC	1095 ± 143*a	73.28 ± 6.75*a	26.71 ± 6.75*a
LAV + SPX	1290 ± 127×a	76.66 ± 8.59×a	23.16 ± 8.54×a
LAV + SPX + NIC	1019 ± 95×a c	70.40 ± 6.73×a	29.60 ± 6.73×a

Data are mean ± SD in the Sham (n = 5), SPX (n = 5), LAV (n = 6), LAV + NIC (n = 6), LAV + MLA + NIC (n = 6), LAV + SPX (n = 6), and LAV + SPX + NIC (n = 6) groups

*LAV* Lavage; *NIC* Nicotine; *SPX* Splenectomy; *MLA* Methyllycaconitine

*: *P* < 0.05, versus the Sham group; **#**: *P* < 0.05, versus the LAV group; a: *P* < 0.05, versus the LAV + NIC group; c: *P* < 0.05, versus the LAV + SPX group; ×: *P* < 0.05, versus the SPX group

## Discussion

The aim of this study was to investigate the role of α7nAChR in the presence or absence of spleen in a sterile model of lung injury induced by repeated saline lavage. We assessed blood pressure, heart rate, airway pressures, gas exchange through blood-gas barrier, lung compliance, immune cells in the blood and BAL, lipid peroxidation, indexes of pulmonary vascular permeability of [Pr] BAL/[Pr] plasma ratio and lung W/D weight ratio, and lung histological score. We used an α7nAChR agonist and a selective α7nAChR antagonist and compared the above parameters in the presence and absence of spleen. We found that α7nAChR plays a significant role in the modulation of inflammation in the saline lavage model of lung injury. Furthermore, stimulating the noted receptor could alleviate some parameters of lung injury, even in the absence of the spleen, whereas the inhibition of it had the opposite effects.

Similar alterations in MBP and HR during saline lavage caused animals to enter the study under equal conditions. Furthermore, the reproducibility of our model is comparable with other studies [25, 26]. Reduction in MBP by saline lavage is analogous with some studies [36] and different from the others [37]. This diversity could be due to the difference in experimental protocols. In our study, the duration of saline lavage and the volume of saline used were adequately high to establish a surfactant-depleted model of lung injury. Furthermore, we injected saline (3 ml/hour, i.p) to maintain the body fluid in all groups of experiments. As a result, any reduction in MBP may not be due to low plasma volume and could be related to hypoxia induced vasodilation [38, 39].The systemic blood pressure decreases in patients with acute respiratory failure under mechanical ventilation. However, the mechanism of hypotension in human ARDS is much more complicated [40].

Moderate increases in MIP and MEP in the Sham and SPX groups are related to mechanical ventilation with PEEP of 4 Cm H_2_O. The progressive increases in MIP and MEP in all LAV groups could be due to the injection of saline and inflammation in the airways. The accumulation of secretions and airway collapse occur in patients with ARDS [41, 42]. The pressure-controlled mechanical ventilation is repeatedly used in the saline lavage model [36, 37]. However, in many studies, PEEP and PIP elevate enormously, which may harm the lungs because of barotrauma [43]. Consequently, we used a volume-controlled ventilation with normal tidal volume to diminish mechanical lung damage in the study.

The evaluation of blood gas parameters indicated severe hypoxemia, moderate hypercapnia, and acidosis in all lavage groups during the recovery phase comparable with data in other studies [36, 37, 44]. Nicotine could correct alterations of PaO_2_, PaO_2_/FiO_2_ ratio, and PaCO_2_ in the LAV + NIC group, whereas MLA has the opposite effect at 3 h after the recovery phase. However, the difference between the LAV + MLA + NIC and LAV + NIC groups was not remarkable which indicates nicotine to some extent improves gas exchange through mechanism linked to α7nAChR. Whether or not other nicotine receptors mediate these alterations remains to be investigated. On the other hand, nicotine increased the systemic blood pressure in our study. It may also increase the pulmonary blood pressure and thereby equalize distribution of blood flow in the lung. As a result, gas exchange can be affected in such a manner. However, we did not measure pulmonary blood pressure or distribution of blood flow in this study which can be suggested in future studies. Splenectomy could not affect gas exchange in the LAV + SPX group compared to the LAV group. Furthermore, nicotine did not improve gas exchange in the LAV + SPX + NIC group. These data indicate splenectomy inhibits gas exchange even in the presence of nicotine and suggests the role of spleen at improving gas exchange in lavage model of lung injury.

The decrease in lung compliance in the LAV group was partially improved by nicotine in deflation volumes of 12 and 14 ml, whereas MLA prevented the lung compliance in deflation volume of 14 ml, which indicates the role of α7nAChR at improving the lung compliance in lung injury. Splenectomy decreased lung compliance in deflation volumes of 12 and 14 ml and nicotine increased lung compliance in deflation volume of 14 ml in the LAV + SPX + NIC group. These results suggest that nicotine could improve the lung compliance through α7nAChR even in the absence of spleen.

We evaluated the effects of α7nAChR agonist and antagonist on MBP and HR. Both parameters increased by nicotine in the LAV + NIC group during the recovery phase. It is important to mention that nicotine could increase MBP in parallel with increased HR. In line with our results, nicotine and some of its metabolites are reported to increase blood pressure together with increased heart rate in anesthetized rats [45]. Also, nicotine increases blood pressure and induces bradycardia in a short time and a tachycardia in a longer time [46]. MLA prevented the increase in MBP in the LAV + MLA + NIC group compared with the LAV + NIC group. These results suggest that nicotine may act through α7nAChR on vessels which is prevented by MLA. However, our conclusion needs more studies for approval. In addition, studies demonstrated the role of α7 and α4β2 nicotinic receptors in cardiovascular sympathetic and parasympathetic activities, respectively [47]. As a result, lower MBP in the LAV + MLA + NIC group compared with the LAV + NIC group may be related to decreased α7-mediated sympathetic activity and/or increased α4β2-mediated parasympathetic activity. On the other hand, MLA did no change HR in the LAV + MLA + NIC group compared with the LAV + NIC group. As a result, nicotine may act on the heart through other nicotinic receptors which cannot be inhibited by the selective antagonist of α7 MLA. In line with our result, it has been indicated that heart rate decreases through activation of α7 subunits of nicotine receptors in a short time, whereas it increases by β4 subunits nicotinic receptors in longer time [48]. It should be emphasized that nicotine increased MBP and HR to the levels in the Sham group. Therefore, it could not disturb the measurements of other parameters in our study.

MBP or HR did not change during the recovery phase in the LAV + SPX group. It should be noticed that we performed splenectomy one week before saline lavage to alleviate the impact of unwanted stresses in the study. Nevertheless, the impact of surgery on cardiovascular system and the elimination effect of spleen as a reservoir in the systemic circulation could still be possible. In addition, it can be suggested that the relation between the pre-ganglionic nicotinic receptors and the sympathetic fibers of the spleen is removed in splenectomized animals. Nicotine as an α7nAChR agonist could not increase MBP and HR in the LAV + SPX + NIC group compared with the LAV + SPX group which may suggest α7nAChR to regulate systemic arterial pressure through spleen. Our hypothesis is supported by a study that the vagus-splenic nerve connection contributes to the regulation of blood pressure linked to T-cell activity and α7nAChR. This idea was demonstrated in coeliac vagotomy and mice lacking α7nAChR subjected to hypertensive challenges [49].

We measured inspiratory and expiratory airway pressures to estimate the airway resistance in the lung. The higher airway pressure means the higher airway resistance. In the recovery phase, nicotine decreased MIP and MEP in the LAV + NIC group, whereas, MLA did not have additional effect in the LAV + MLA + NIC group. The same reductions in MIP and MEP in the LAV + NIC and LAV + MLA + NIC groups suggests that these alterations cannot be ascribed only to the effect of α7nAChR. Splenectomy did not change the above parameters neither in the LAV + SPX group, nor in the LAV + SPX + NIC group. It seems that the effect of nicotine on airways is independent of spleen. Inhaled cigarette smoke and intravenous nicotine injection induce bronchoconstriction in dogs through medullary and peripheral nicotine receptors [50]. Even inhaled nicotine with concentrations found in e-cigs causes significant pulmonary damage [51]. However, we used a very low dose i.p injection of nicotine in our study similar to other studies [52]. The low dose nicotine may lead to bronchodilation through the activation of preganglionic neurons of the sympathetic system innervating the lower airways via beta-2 adrenergic receptors likewise to that seen in the cardiovascular system [53]. Furthermore, the effect of nicotine on the airways could be related to surfactant secretion because nicotine stimulates the expression of surfactant proteins of SP-A and SP-C mRNAs in embryonic mouse lung culture [54]. Also, the cholinergic stimulation of surfactant secretion is linked to type 2 alveolar cells [55].

In order to evaluate inflammatory reactions, we measured total and differential WBC in the blood and BAL [29]. Total WBC in the blood decreased in the LAV group, whereas nicotine increased it in the LAV + NIC group. As a result, nicotine has anti-inflammatory effect in the presence of spleen. In addition, the decrease in neutrophils % and the increase in lymphocytes % in the LAV group were corrected by nicotine, whereas MLA had the opposite effects. These data confirm the anti-inflammatory effect of nicotine through α7nAChR. Also, total WBC in the blood decreased in LAV + SPX group, whereas nicotine prevented this reduction. Nicotine returned partially the neutrophils % and lymphocytes % in the LAV + SPX + NIC group, which may suggest nicotine alleviates inflammation partly through preventing the migration of  leukocytes to the injured lung. The above hypothesis could be confirmed by the results of BAL cell count. Total BAL cells increased in the LAV group, attenuated by nicotine, and reversed by MLA. Increased neutrophils % in the LAV group suggests neutrophil migration from the blood to the lung. Nicotine decreased neutrophils %, and MLA increased it. The macrophages % in the BAL decreased because the number of neutrophils in the BAL increased. Macrophages % increased by nicotine in the LAV + NIC group, and decreased by MLA. These results indicates the role of α7nAChR in preventing inflammation in the lung. Total BAL cells, neutrophils %, and macrophages % in the LAV + SPX group were similar to those in the LAV group, whereas nicotine only partly corrected them, which indicates even in the presence of nicotine, inflammation remains in the absence of spleen.

We measured MDA in plasma and lung to evaluate the oxidative stress in our study. We found nicotine to decrease in plasma and lung tissue MDA in the LAV + NIC group. The increase in MDA was prevented by MLA in the LAV + MLA + NIC group which obviously clarify the role of α7nAChR in modulating the oxidative stress in our model of lung injury. On the other hand, splenectomy exacerbated oxidative stress in plasma and lung tissue. However, nicotine could partially reduce lung MDA in the LAV + SPX + NIC group, which suggests incomplete effect of nicotine through α7nAChR in decreasing the oxidative stress in the lung.

We measured [Pr] BAL/[Pr] plasma ratio and W/D weight ratio to evaluate pulmonary vascular permeability. Lung W/D ratio increased in the LAV group, attenuated by nicotine, and reversed by MLA, which suggests the role of α7nAChR in reducing vascular permeability in lung injury. Splenectomy increased lung W/D ratio in the LAV + SPX group which inhibited partially by nicotine. Results of [Pr] BAL/[Pr] plasma ratio were almost similar to the results of lung W/D ratio. But the effects of nicotine in the LAV + MLA + NIC and LAV + SPX + NIC groups were not significant. The same results as [Pr] BAL/[Pr] plasma ratio were obtained from data of lung histological score, the indicator of lung tissue damage. Together, our results indicate at least a part of increased vascular permeability cannot be repaired in the splenectomized animals even in the presence of α7nAChR agonist.

## Conclusions

In this study, we indicated roles of α7nAChR and spleen in alleviating the inflammation in the non-pathogenic model of lung injury. We found that the systemic arterial blood pressure, heart rate, gas exchange through blood gas barrier, inflammation, pulmonary vascular permeability, oxidative stress and lung tissue injury could be regulated by α7nAChR separately and accompanied by spleen. However, lung compliance and airway pressure are controlled by nicotine through α7nAChR even in the absence of spleen. Whether or not other subunits of nicotine receptors play a role needs to clarify in future studies.

### Limitation of the study

Repeated saline lavage has long been used as a reproducible pathogen-free model of lung injury in animals. The lung injury is established rapidly and will be stable until 10 h [25, 28]. However, the saline lavage must be done under deep anesthesia and mechanical ventilation. Therefore, this lung injury model cannot be used in long term studies [27]. Moreover, human ARDS can be affected by primary diseases, therapeutic approaches, and hereditary factors that cannot be considered in this model [56]. As a results, this model similar to other animal models of lung injury may not completely mimic all pathophysiological features of lung injury in human.

## Data Availability

Our data will be available from the corresponding author on reasonable request.

## Abbreviations

ACh: Acetylcholine
α7nAChR: α7 Nicotinic Acetylcholine receptors
ANOVA: One-way analysis of variance
ARDS: Acute respiratory distress syndrome
BAL: Bronchoalveolar lavage
CAP: Cholinergic anti-inflammatory pathways
FiO_2_: Fraction of inspired oxygen
HR: Heart rate
LAV: Lavage
LPS: Lipopolysaccharide
NIC: Nicotine
MBP: Mean arterial blood pressure
MDA: Malondialdehyde
MEP: Maximum expiratory pressure
MIP: Maximum inspiratory pressure
MLA: Methyllycaconitine
PaO_2_: Arterial oxygen pressure
PaCO_2_: Arterial carbon dioxide pressure
PEEP: Positive end expiratory pressure
[Pr]: Plasma protein concentration
SPX: Splenectomy
TBARS: Thiobarbituric acid reactive substances
TLR: Toll like receptor
